# Digital performance testing reveals how redesigned return cannulas can enhance VV-ECMO performance

**DOI:** 10.1186/s40635-026-00927-z

**Published:** 2026-06-15

**Authors:** Beata Ondrusova, Argyrios Petras, Johannes Szasz, Jens Meier, Luca Gerardo-Giorda

**Affiliations:** 1 Institute for Mathematical Methods in Medicine and Data Based Modeling, Johannes Kepler University, Linz, Austria; 2 Johann Radon Institute for Computational and Applied Mathematics (RICAM), Austrian Academy of Sciences, Linz, Austria; 3 Kepler University Klinikum, Johannes Kepler University, Linz, Austria

**Keywords:** Veno-venous extracorporeal membrane oxygenation, Computational fluid dynamics, Recirculation fraction, Hemolysis, Thrombosis

## Abstract

**Background:**

Veno-venous extracorporeal membrane oxygenation (VV-ECMO) provides life support for patients with severe respiratory failure by oxygenating blood when the lungs are impaired. Standard VV-ECMO uses two cannulas to drain and return blood. However, recirculation, where oxygenated blood is immediately withdrawn back into the circuit by the drainage cannula, reduces effective oxygen delivery. With survival rates around 62%, optimizing VV-ECMO is essential to improve patient outcomes.

**Methods:**

In this study, computational modeling was used to investigate recirculation, oxygen delivery, and the risk of thrombosis and hemolysis for six different return cannula designs under three ECMO flow rates (2, 4, and 6 L/min). Two commercially available and four novel cannulas were tested to evaluate whether design modifications could improve oxygen delivery. Among the novel designs, one was open with additional holes, while the others featured closed or perforated caps.

**Results:**

At low ECMO flow (2 L/min), all cannulas showed similar oxygen saturation (78%) and low recirculation (1.6%), indicating minimal impact of cannula design on VV-ECMO performance. At high flow (6 L/min), closed-tip cannulas reduced recirculation by 40–50% and increased oxygen saturation by 6%, without substantially affecting the risk of thrombosis and hemolysis. Moreover, the partial pressure of oxygen increased by up to 151% compared to the cannula commonly used in clinical practice.

**Conclusion:**

Our findings suggest that new return cannula design could enhance VV-ECMO performance, especially in patients relying predominantly on the support due to severely impaired lung function.

**Supplementary Information:**

The online version contains supplementary material available at 10.1186/s40635-026-00927-z.

## Backround

Recirculation is a key limitation of veno-venous extracorporeal membrane oxygenation (VV-ECMO) and refers to the phenomenon in which fully oxygenated blood delivered to the patient through the return cannula is immediately drawn back into the ECMO circuit via the drainage cannula, thereby reducing effective oxygen delivery [1] and potentially resulting in hypoxemia despite ongoing ECMO support [2]. Typically, recirculation is expressed as the recirculation fraction (%), defined as the proportion of ECMO flow that is recaptured by the drainage cannula and returned to the ECMO circuit [3]. Recirculation is commonly estimated using oxygen saturation-based formulas incorporating pre-oxygenator, post-oxygenator, and systemic venous oxygen saturation [4], or alternatively by using ultrasound dilution [5]. The typical recirculation fraction observed in clinical practice is below 20% [6, 7], but it can reach values as high as 58% [6].

Recirculation is observed particularly in classical double cannulation strategies when the cannula tips are positioned too close to each other or when the tip of the return cannula is oriented toward the inlet of the drainage cannula [4, 7]. Repositioning of ECMO cannulas to decrease recirculation can be challenging, as it carries risks of vascular injury and bleeding [8], requires imaging guidance [9], and may require an individualised approach based on patient status [10]. Moreover, many other parameters, such as the ratio between cardiac output and ECMO flow [11], as well as cannula design [5], were identified as the factors influencing the recirculation fraction. Furthermore, patients receiving VV-ECMO are at risk of several complications. The most common is bleeding, which is strongly associated with increased mortality [12]. Thrombotic events are also frequent, with circuit-related thrombosis being the most prevalent [12]. Hemolysis, although less common, is clinically significant, because it can lead to renal dysfunction and tissue hypoxia [13].

While in vitro and animal studies have long guided ECMO development [14], computational fluid dynamics (CFD) now enables detailed, non-invasive analysis of blood flow. Ongoing CFD research has advanced the understanding and optimisation of VV-ECMO cannula placement and design. Studies show that recirculation increases as the distance between cannula tips decreases, though this does not necessarily impair oxygenation efficiency [15]. Furthermore, it allows to study the interaction between native cardiac output and ECMO flow within a controlled CFD framework, which cannot be achieved at the bedside [16, 17]. CFD also enables the evaluation of current cannula designs and configurations [18, 19], and the development of new designs [20]. Moreover, the use of patient-specific geometries allows for non-invasive investigation of regions with high shear stress, recirculation, or flow stagnation, which are associated with adverse clinical outcomes [18, 21, 22].

The substantial burden of complications, together with survival rates of 62% [23], highlights the need for continued optimisation of VV-ECMO systems to improve patient outcomes. In this work, we use CFD modelling to study the level of recirculation and oxygen saturation in a patient-specific VV-ECMO model using a femoro-jugular cannulation strategy with a pressure-driven tricuspid valve boundary condition. This study evaluates six return cannula designs under three ECMO flow rates, including two widely used commercially available single-lumen cannulas and four novel performance-driven designs, to assess their impact on VV-ECMO effectiveness. Complications such as thrombosis and hemolysis are multifactorial processes and cannot be directly predicted using CFD alone, and no universally accepted methodology exists for their assessment using CFD. Nevertheless, simulation-derived parameters such as endothelial cell activation potential (ECAP) [24] and the index of hemolysis (IH) [25] are used in this study as surrogate markers to estimate the associated risk in comparison with a standard model of returning cannula. This study demonstrates how CFD can support evidence-based optimisation of extracorporeal circuits.

## Materials and methods

### Data

**Fig. 1:**
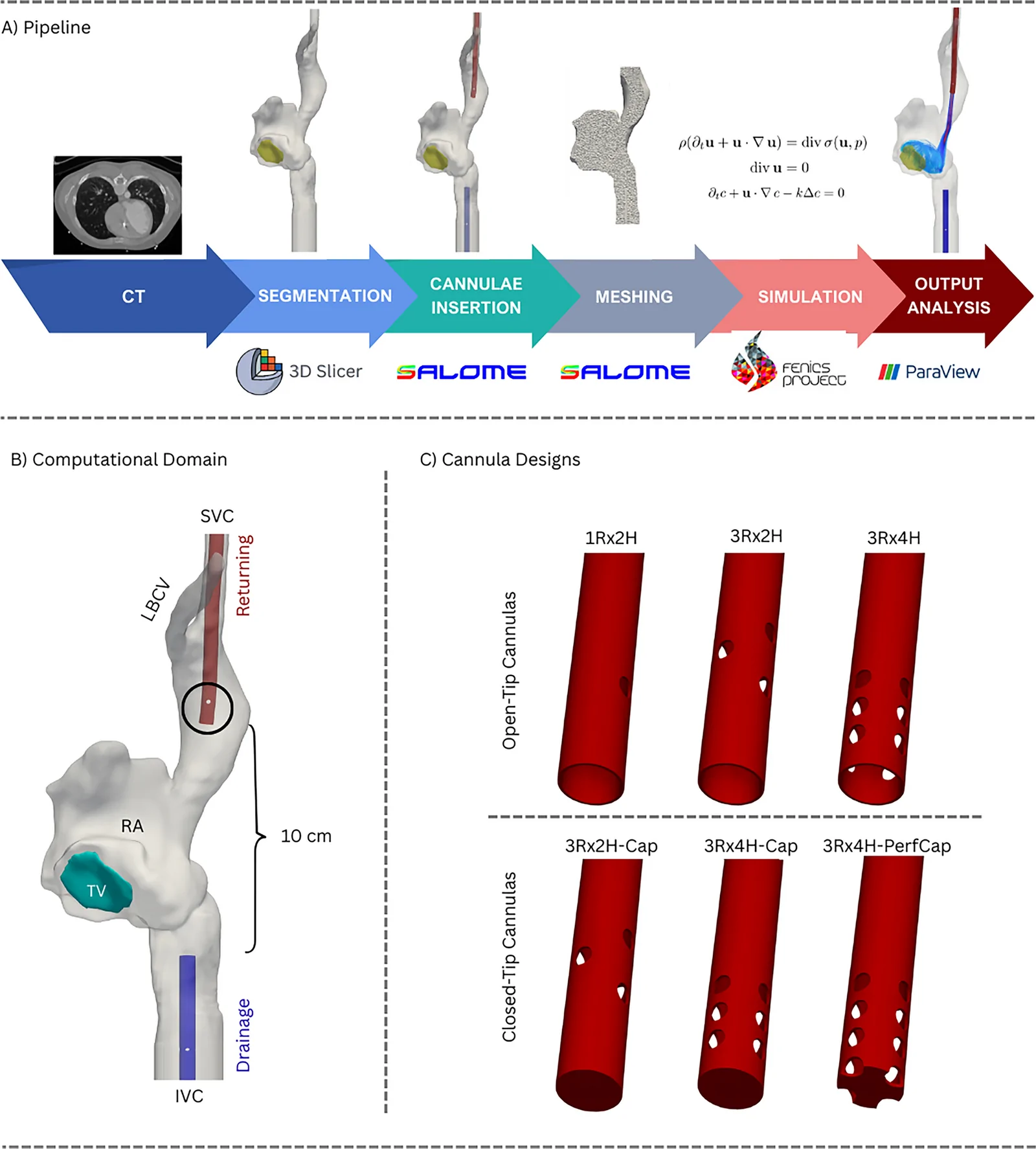
**A** Schematic overview of the study pipeline, illustrating the workflow and computational tools used. **B** Posterior coronal view of the computational domain illustrating the main anatomical structures and cannula positions. The only difference among the configurations is in the design of the return cannula tip, highlighted by the black circle. **C** Designs of the return cannulas categorised as open-tip and closed-tip cannulas. Models 1Rx2H and 3Rx2H are commercially available; the remaining designs are conceptual designs. SVC : Superior Vena Cava, LBVC: Left Brachiocephalic Vein, RA: Right Atrium, TV: Tricuspid Valve, IVC: Inferior Vena Cava

The pipeline of this study is shown in Fig. 1A. The patient-specific geometrical model, including the right atrium (RA), superior vena cava (SVC), left brachiocephalic vein (LBCV), inferior vena cava (IVC) and tricuspid valve (TV), was created by segmenting a contrast-enhanced CT scan of a male patient using 3D Slicer [26]. Anonymised imaging data were provided by Kepler Klinikum (Linz, Austria) with informed patient consent. No additional patient-specific information was used in this study. All imaging data handling complied with institutional regulations and adhered to the ethical principles of the Declaration of Helsinki.

### Cannula designs

In this study, we investigated the femoral–jugular cannulation strategy, which is commonly used in clinical practice [27]. The drainage cannula, inserted into the IVC, and the return cannula, positioned in the SVC, were modelled using the Salome software [28]. The distance between the tips of both cannulas was set to 10 cm, reflecting common clinical practice [29] as shown in Fig. 1B. The drainage cannula design was based on the Maquet BE-PVL 2155 (21 Fr) and remained unchanged across all simulations. In contrast, six return cannula designs were modelled and evaluated. Each design of returning cannula is illustrated in Fig. 1C. The return cannula models differ only in the number and arrangement of side holes and the design of the distal cap, while the insertion path and outer diameter (19 Fr) remain constant. Two of the six designs were based on commercially available models, while the remaining four were experimental designs proposed to observe differences in VV-ECMO efficacy:

- 1Rx2H – Inspired by the Maquet BE-PAL 1923 cannula (1 row with 2 side holes) [30].
- 3Rx2H – Inspired by the Medtronic Flex dual-lumen cannula (3 rows with 2 holes each) [31].
- 3Rx4H – Adapted from 3Rx2H with an increased number of side holes (3 rows with 4 holes each).
- 3Rx2H-Cap – The 3Rx2H model with a closed-tip distal cap.
- 3Rx4H-Cap – The 3Rx4H model with a closed-tip distal cap.
- 3Rx4H-PerfCap – The 3Rx4H-Cap model with a perforated distal cap (4 regularly spaced holes between the cap and the cannula wall).

In order to clearly identify each design, a feature-based naming convention was adopted: the notation indicates the number of rows followed by the number of holes per row (e.g., "3Rx2H" corresponds to 3 rows with 2 holes each), along with suffixes denoting distal tip modifications, such as a closed-tip cap ("-Cap") or a perforated cap ("-PerfCap"). Furthermore, for discussion purposes, we group models 1Rx2H, 3Rx2H, and 3Rx4H as "open-tip cannulas," and the rest as "closed-tip cannulas". The side hole positions are placed, such that the holes in the 1Rx2H model form a subset of those in the 3Rx2H model, which in turn are a subset of the holes in the 3Rx4H model. Thus, the positions of the side holes remain comparable across all models and eliminating the influence of hole placement as a variable.

### Mathematical modeling

The blood flow within the patient-specific model was computed by solving the incompressible Navier–Stokes equations, which govern the conservation of mass and momentum. The model used a blood density of $\rho = 1050\, \mathrm {kg/m^3}$ and a dynamic viscosity of $\mu = 3.65 \times 10^{-3}\, \mathrm {kg/(m \cdot s)}$ [32]. A single patient-specific anatomical geometry was used to generate six computational domains, differing only in the geometry of the return cannula, while the drainage cannula remained unchanged. Seven boundaries were defined: inlets at the SVC, LBCV, IVC, and return cannula; outlets at the TV and drainage cannula; and walls. The simulation was initialised with zero velocity for both cannulas. Following a brief initialisation period of 4 s, a steady parabolic velocity profile corresponding to the ECMO flow was imposed at the return cannula inlet and the drainage cannula outlet was modelled with the same velocity profile. Time-dependent pressure boundary conditions were imposed at the SVC, LBCV and IVC inlets to prevent unphysiological backflow. The outlet of the TV was modelled as a dynamic boundary that alternated between closed (no flow) and open states, controlled by a time-varying pressure difference based on the Wiggers diagram [33]. This approach allowed for a realistic simulation of the valve’s function in regulating flow from the RA to the right ventricle (RV). Vessel and cannula walls were treated as rigid surfaces with no-slip conditions, ensuring zero velocity at all solid boundaries.

Oxygen transport was simulated by solving an advection–diffusion equation. Advection was driven by the blood velocity field. Fully oxygenated blood (saturation of 100%) was defined at the return cannula inlet, while deoxygenated blood (saturation of 70%) was set at the SVC, LBCV and IVC inlets. Oxygen exchange across the vessel walls was neglected, assuming these boundaries to be impermeable to gas transfer. For more details about the modelling approaches, please refer to Leoni et al. [15].

### Simulation scenarios

To ensure the physiological relevance of this study, all simulation parameters were based on representative clinical conditions. Accordingly, three setups derived from clinical data reported by Loosen et al. [34] were used to investigate different levels of VV-ECMO support using mean values, as summarised in Table 1. These setups represent distinct hemodynamic states. The extracorporeal flow fraction (EFF) was computed as a ratio of ECMO flow to cardiac output (CO). The ’Partial Support’ setup represents a condition in which the VV-ECMO circuit provides limited assistance with gas exchange, with the EFF of 26%, indicating that native lung function remains the primary source of oxygenation. The "Intermediate support" reflects a scenario in which VV-ECMO contributes more substantially to gas exchange with an EFF of 62%, partially offloading the lungs. The "Full Support" setup describes a state in which VV-ECMO provides the majority of gas exchange, effectively relieving the native lungs of their function.

**Table 1:** Simulation setups based on clinical data from Loosen et al. [34]

Setup	ECMO flow (L/min)	HR(bpm)	CO (L/min)	EFF (%)
Partial support	2	76	7.6	26
Intermediate support	4	76	6.5	62
Full support	6	73	6.0	100

EFF was computed as the ratio of ECMO flow to CO. HR: Heart rate, CO: cardiac output, EFF: extracorporeal flow fraction

### HPC implementation

A custom-built FEniCSx solver [35] was used to perform all simulations on the Radon1 HPC cluster at the Johann Radon Institute for Computational and Applied Mathematics (RICAM) in Linz, Austria [36]. Each simulation was executed over a 60-s interval, with results recorded during the final 30 s to ensure steady-state conditions.

### VV-ECMO efficacy

The recirculation fraction ($R_F$), oxygen saturation (${SO}_2$), arterial oxygen content (${CaO}_2$), and partial pressure of oxygen (${PO}_2$) were computed to study VV-ECMO effectiveness for each return cannula design and ECMO flow. The analysis was performed for all heartbeats recorded during a 30-s window, with results averaged over those heartbeats.

The recirculation fraction $R_F$ (%) was calculated as the average percentage of oxygen above the baseline level that flows out through the outlet of the drainage cannula as

1 $$\begin{aligned} R_f = \frac{1}{t_{end} - t_{start}} \int _{t_{start}}^{t_{end}} \frac{1}{ECMO_{flow}} \int _{C_{drain}} {cu} \cdot {n} \, ds \, dt. \end{aligned}$$

The inner integral computes the instantaneous flux of oxygen through the outlet at a given time, where $C_{drain}$ is the drainage cannula outlet, *c* is the local oxygen concentration, *u* is the blood flow velocity vector, *n* is the unit normal vector, and *ds* is the surface area element. The outer integral computes the time-averaged value, where $t_{\mathrm{start}}$ and $t_{\mathrm{end}}$ represent the time interval, and $\mathrm{ECMO}_{\mathrm{flow}}$ corresponds to the values in Table 1.

The average oxygen saturation $SO_2$ (%) at the TV was estimated using formula:

2 $$\begin{aligned} {SO}_2=70+30e, \end{aligned}$$

where *e* is the effectiveness ranging from 0 to 1. Effectiveness *e* reflects the fraction of oxygenated blood passing through the TV, where 0 means only venous blood (70% saturation) and 1 means fully oxygenated blood (100% saturation). Effectiveness *e* was calculated for each heartbeat by integrating the oxygen content and total blood flow through TV over time using numerical integration. Further details on the evaluation of VV-ECMO efficacy in terms of $R_F$ and $SO_2$ can be found in Leoni et al. [15].

Arterial oxygen content $CaO_2$ (mL/dL) was estimated as

3 $$\begin{aligned} CaO_2 = 1.36 \times 12 \times (SO_2/100), \end{aligned}$$

where the constant 1.36 represents Hüfner’s number in mL/g, the multiplication by 12 assumes a haemoglobin concentration of 12 g/dL and $SO_2$ was derived from simulations [37]. Dissolved oxygen was neglected due to its comparatively small contribution to total oxygen content.

The partial pressure of oxygen $PO_2$ (mmHg) was derived using inversion of Hill’s equation as

4 $$\begin{aligned} \mathrm{P}{\mathrm{O}_2}^n = P50^n \times \left( \frac{\mathrm{SO}_2}{1-\mathrm{SO}_2} \right) , \end{aligned}$$

where *P*50 represents the oxygen partial pressure at 50% hemoglobin saturation (27 mmHg), and *n* is the Hill coefficient, which was set to 2.8.

### Risk prediction

Thrombosis and hemolysis are associated with increased mortality and other complications in patients on VV-ECMO; therefore, we evaluated the risks of these adverse outcomes for each cannula design across all ECMO flows.

#### Thrombosis

To assess the risk of thrombosis, four haemodynamic parameters were evaluated, as described in [24]. Time-averaged wall shear stress (TAWSS) was computed as the temporal average of the magnitude of wall shear stress over one cardiac cycle as

5 $$\begin{aligned} \mathrm{TAWSS} = \frac{1}{T} \int _0^T |\vec {\tau }_w(t)| \, dt, \end{aligned}$$

where $\vec {\tau }_w(t)$ is the instantaneous wall shear stress vector. Oscillatory shear index (OSI) was calculated to quantify the directional changes of wall shear stress as

6 $$\begin{aligned} \mathrm{OSI} = \frac{1}{2} \left( 1 - \frac{\left| \int _0^T \vec {\tau }_w(t) \, dt \right| }{\int _0^T |\vec {\tau }_w(t)| \, dt} \right) . \end{aligned}$$

The values of OSI range from 0 for unidirectional flow to 0.5 for highly oscillatory flow. Relative residence time (RRT) was computed to identify areas of low and oscillatory flow as

7 $$\begin{aligned} \mathrm{RRT} = {\frac{1}{(1 - 2 \, OSI) \, \mathrm{TAWSS}}}. \end{aligned}$$

Finally, endothelial cell activation potential (ECAP) was computed as

8 $$\begin{aligned} \mathrm{ECAP} = \frac{\mathrm{OSI}}{\textrm{TAWSS}}. \end{aligned}$$

ECAP highlights areas, where oscillatory and low-magnitude shear stress occur, which are prone to thrombosis.

All parameters were computed for the inner wall of the returning cannula as well as for the walls of the veins and the right atrium over one cardiac cycle.

#### Hemolysis

The index of hemolysis (IH) was computed using velocity pathlines and a power law as

9 $$\begin{aligned} \mathrm{IH} = A_{Hb}(\mu G_s)^{\alpha {Hb}}t^{\beta _{Hb}}, \end{aligned}$$

where $A_{Hb}$, $\alpha _{Hb}$, and $\beta _{Hb}$ are empirical coefficients derived by Gierspen [38] with values $3.62 \times 10^{-5}$, 2.416, and 0.785, respectively, μ is the dynamic viscosity of blood that equals to $0.0035~\mathrm {Pa\cdot s}$, $G_s$ is a scalar shear rate computed as proposed by Bludszuweit [39] and *t* is the exposure time. The mean $\textrm{IH}$ was computed for a given cannula design and ECMO flow by averaging over all pathlines.

**Fig. 2:**
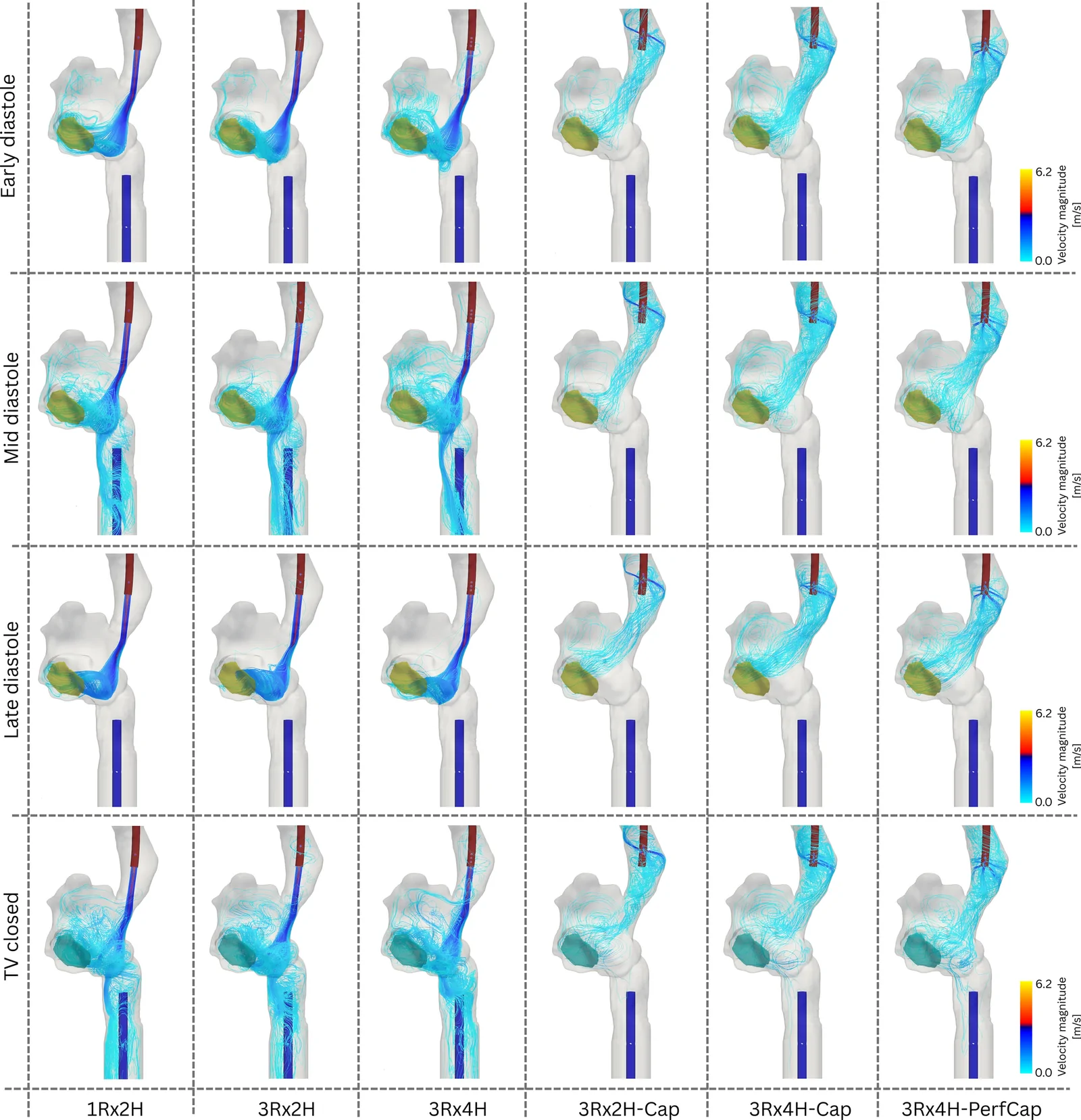
Blood flow from return cannula during different phases of the ventricular cycle (rows) for all cannula designs (columns). The colour of the tricuspid valve indicates the cardiac cycle phase: yellow when open and blue when closed. The results are depicted for the ECMO flow of 4 L/min

Short pathlines, capturing flow inside and slightly outside the returning cannula, as well as pathlines spanning the entire computational domain, were used for analysis. Shorter pathlines were analyzed to isolate the effects of cannula geometry on hemolysis. In addition, pathlines in the entire computational domain computed during ventricular systole were used to capture how flow alterations influence hemolysis. The TV closed condition was chosen, because it produces more turbulent and complex blood flow patterns, whereas with the TV open, blood flows directly through the TV, as shown in Fig. 2. For hemolysis assessment, the HemTracer tool was used as described by Dirkes et al. [25].

## Results

### VV-ECMO efficacy

**Fig. 3:**
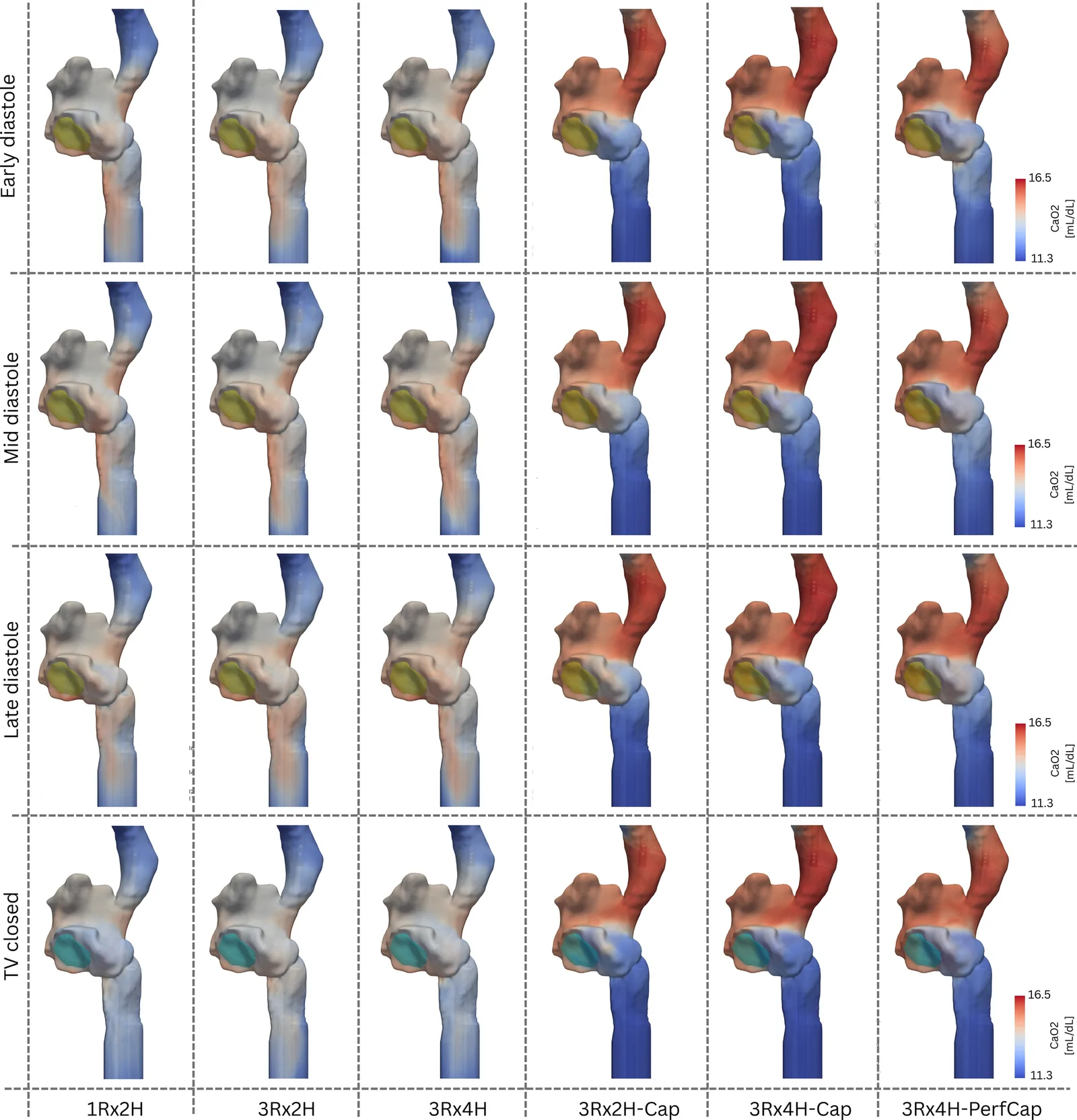
Oxygen content ($CaO_2$) in right atrium during different phases of the ventricular cycle (rows) for all cannula designs (columns). The colour of the tricuspid valve indicates the cardiac cycle phase: yellow when open and blue when closed. The results are depicted for the ECMO flow of 4 L/min

The blood flow from all models of return cannulas is depicted in Fig. 2 and $CaO_2$ content in RA is depicted in Fig. 3. The results are presented for an ECMO flow rate of 4 L/min, corresponding to a flow-to-cardiac output ratio of 62%, a commonly used setting in clinical practice [40]. In addition, the results are shown across different phases of the cardiac cycle to assess pressure-dependent variations in flow dynamics and oxygen diffusion. The figures corresponding to different ECMO flows as well as videos showing flow from the return cannulas and $CaO_2$ are included in the Supplementary Material.

**Fig. 4:**
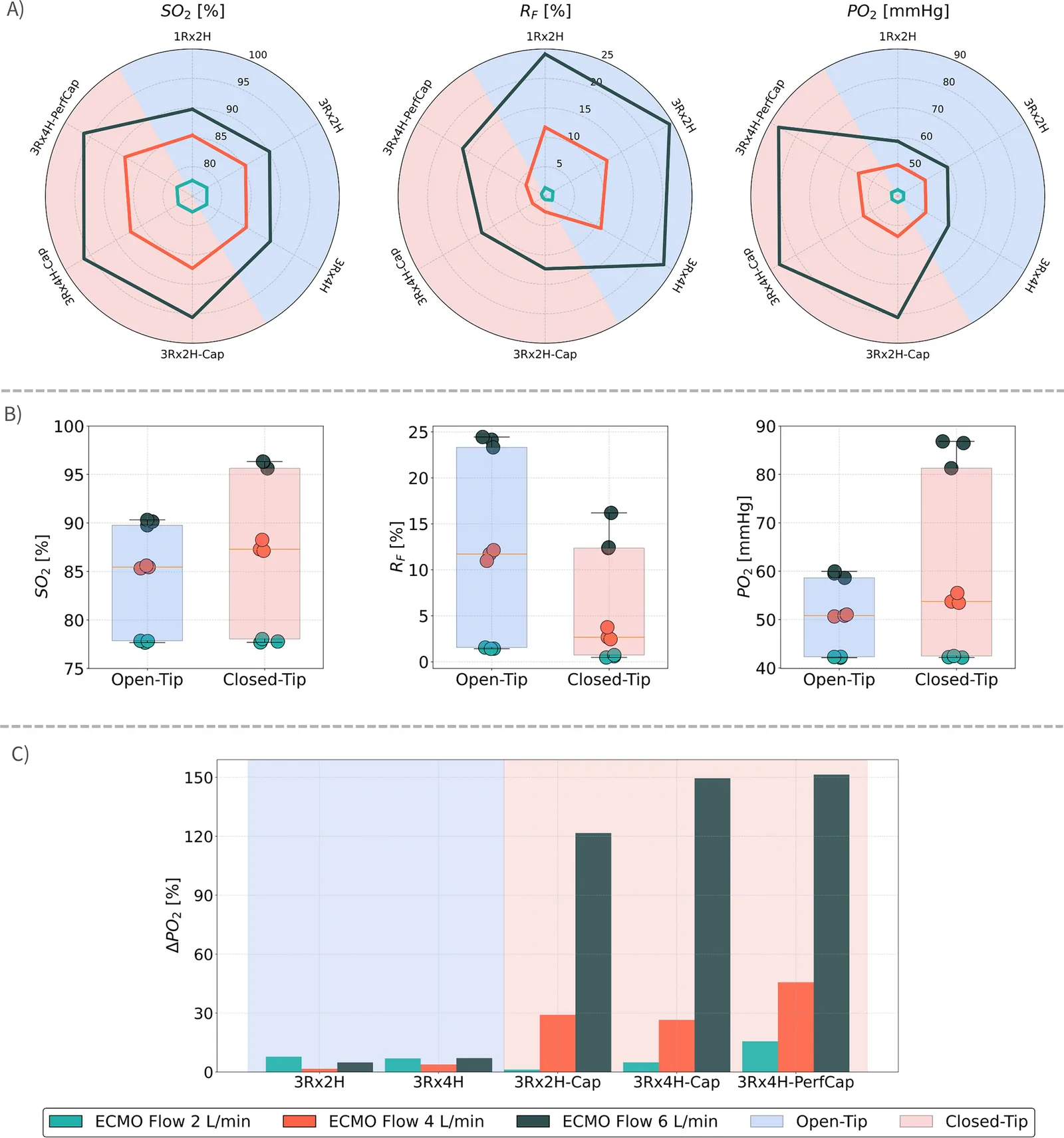
**A** Radar plots depicting mean values of oxygen saturation ($SO_2$), recirculation fraction ($R_F$) and partial pressure of oxygen ($PO_2$). Cannula designs are represented on a separate axis radiating from a central point with different ECMO flows represented by distinct colors. **B** Mean values of oxygen saturation ($SO_2$), recirculation fraction ($R_F$), and partial pressure of oxygen ($PO_2$) for open-tip and closed-tip cannulas (different boxes), across all ECMO flow rates. Scatter points represent individual observations. **C** Percentage increase in $PO_2$ relative to the reference cannula 1R×2 H

Figure 2 suggests that $R_F$ may be lower for closed-tip designs of return cannulas, as the flow is directed toward the TV throughout the entire cardiac cycle. This observation is also supported by numerical results shown in Table 2 and Fig. 4A, which presents $SO_2$, $R_F$, and $PO_2$ for all ECMO flow rates and return cannula designs. The lowest $SO_2$, $PO_2$, as well as $R_F$ were observed with ECMO flow of 2 L/min. The highest $SO_2$ and $PO_2$ values were observed at an ECMO flow rate of 6 L/min, mainly with closed-tip cannulas, which also demonstrated a reduction in $R_F$ compared to open-tip cannulas.

**Table 2:** Mean values of oxygen saturation ($SO_2$), recirculation fraction ($R_F$) and partial pressure of oxygen ($PO_2$) for all cannula designs and ECMO flows

Parameter	Flow (L/min)	1Rx2H	3Rx2H	3Rx4H	3Rx2H-Cap	3Rx4H-Cap	3Rx4H-PerfCap
$SO_2$ (%)	2	77.7	77.9	77.8	77.7	77.8	78.0
	4	85.3	85.5	85.6	87.3	87.1	88.3
	6	89.8	90.2	90.3	95.6	96.3	96.3
$R_F$ (%)	2	1.4	1.6	1.4	0.6	0.5	0.7
	4	11.7	12.1	11.0	2.7	2.5	3.8
	6	24.2	24.4	23.3	12.4	12.4	16.2
$PO_2$ (mmHg)	2	42.1	42.3	42.3	42.2	42.2	42.5
	4	50.6	50.8	51.0	53.7	53.5	55.5
	6	58.6	59.5	59.9	81.3	86.5	86.8

Figure 4B presents a comparative analysis of open-tip vs. closed-tip cannula designs and shows the statistical distributions of $SO_2$, $R_F$ and $PO_2$ values across all ECMO flow rates. In all cases, the closed-tip cannulas demonstrate improved performance, with the most distinctive improvement observed at 6 L/min.

Finally, we compared the performance of all cannulas to the 1Rx2H cannula, a standard clinical design with the fewest side holes among those evaluated in this study. The results are summarised in Fig. 4C. We calculated the percentage increase in $PO_2$ above a venous baseline of 40 mmHg for each cannula, normalised by the corresponding $PO_2$ increase observed with the 1Rx2H cannula. It is noticeable that open-tip cannulas with more side holes exhibit similar performance compared to cannula with just 2 side holes, whereas closed-tip cannulas performed better compared to standard designs and the gain in $PO_2$ increases with increasing ECMO flow.

### Risk prediction

The risk of thrombosis and hemolysis is evaluated through CFD-derived metrics, such as ECAP, RRT, and IH, respectively. However, due to the absence of well-defined threshold values for predicting thrombosis and hemolysis from CFD studies, the performance of all cannulas is evaluated relatively to the standard cannula model (1Rx2H). Results are expressed as normalised ratios, where values bigger than 1 indicate an increase in the metric with respect to the standard cannula, while values smaller than 1 indicate a decrease. The results are presented for the cannulas themselves as well as for the walls of the computational domain, which includes the veins and right atrium.

**Fig. 5:**
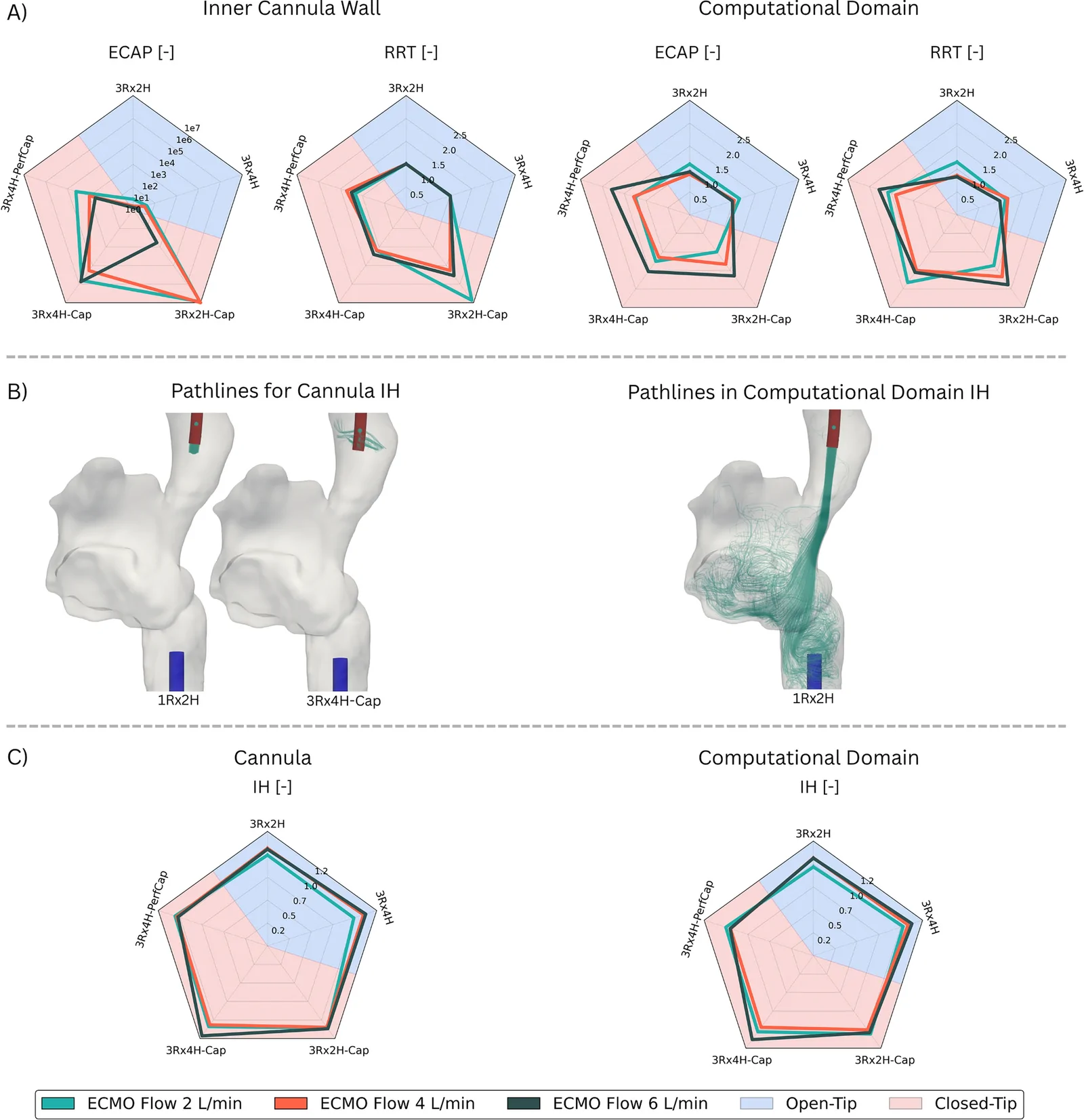
A Radar plots depicting the values of endothelial cell activation potential ($\textrm{ECAP}$) and relative residence time ($\textrm{RRT}$) normalized with respect to standard cannula 1Rx2H. ECAP and RRT were computed for the inner wall of the returning cannula and the computational domain. B Example illustration of pathlines used to assess hemolysis risk. Pathlines were computed either from the cannula alone or across the entire computational domain. C Radar plots depicting the index of hemolysis (IH) normalized with respect to standard cannula 1Rx2H. In radar plots, cannula designs are represented on a separate axis radiating from a central point with different ECMO flows represented by distinct colors. Values > 1 indicate an increase, and values <1 a decrease to standard cannula 1Rx2H

**Fig. 6:**
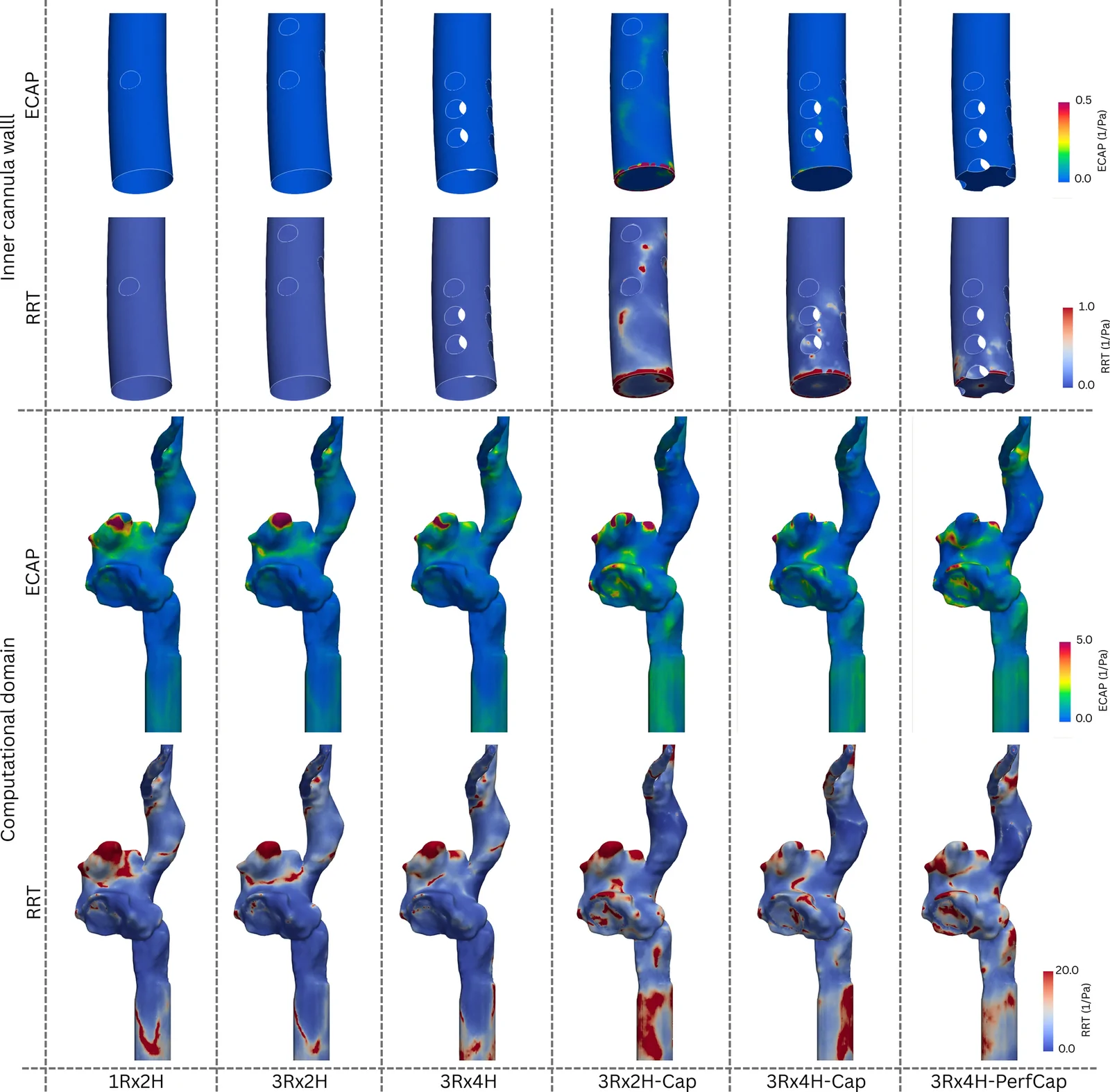
Endothelial cell activation potential (ECAP) and relative residence time (RRT) depicted for the inner wall of the returning cannula and the computational domain consisting of walls of the veins and right atrium. Colorbar limits are set separately for the cannula and the computational domain because of differences in their respective values. The results are depicted for the ECMO flow of 4 L/min

At the inner cannula surface, open-tip designs exhibited negligible variation of ECAP with respect to the standard cannula, whereas closed-tip designs showed increases of several orders of magnitude, as shown in Fig. 5. However, their absolute values remain substantially lower—by approximately one to six orders of magnitude depending on cannula design and ECMO flow rate (see supplementary material)—than those in the whole computational domain (see Fig. 6), where the relative variations are actually small.

Similarly, the risk of hemolysis was assessed for each cannula to determine whether interaction of blood flow with a closed cap could increase hemolysis risk with respect to the standard cannula, both inside the tip and across the entire computational domain. Example pathlines for open- and closed-tip cannulas are depicted in Fig. 5B. As shown in panel 5C, hemolysis risk is not substantially influenced by cannula design.

## Discussion

In this work, we used computational modeling to evaluate the effectiveness of VV-ECMO under different flow rates and different return cannula designs. We tested both commercially available open-tip cannulas and conceptual designs with more holes or closed cap to investigate whether improvements in VV-ECMO performance could be achieved.

The VV-ECMO efficacy for all cannula designs and ECMO flows is summarised in Table 2 and Fig. 4A. The efficacy was assessed in terms of recirculation and oxygen delivery, which serves as the primary indicator of clinical performance. Consistent with previous findings, higher ECMO flow rates are associated with increased recirculation but also result in higher oxygen content, as reflected by increased $SO_2$ and $PO_2$ [15, 17]. However, one can notice a clear distinction between the performance of open-tip and closed-tip cannulas, as shown in Fig. 4B. At low ECMO flow (2 L/min), all cannulas show similar oxygen saturation (78%), low recirculation (1.6%), and comparable $PO_2$ (42 mmHg). The $PO_2$ increase relative to the standard cannula 1Rx2H is minimal for all designs, ranging to 16%, indicating that under low-flow conditions, cannula design has little impact on improvement in systemic oxygen delivery. As ECMO flow increases, the impact of cannula design becomes more pronounced. The differences are most striking at 6 L/min, where oxygen saturation is approximately 6% higher (96% vs. 90%), and recirculation is reduced by approximately 40–50% (12–16% vs. 23–24%) for closed-tip cannulas. Moreover, the closed-tip cannulas exhibit *PO*2 increase of 122–151 % compared to a standard cannula 1Rx2H. The observed differences in $PO_2$ relative to the standard cannula also indicate lower levels of recirculation.

The improvement in VV-ECMO efficacy with closed-tip cannulas can be explained by the streamline visualizations of blood flow from the return cannula as depicted in Fig. 2 for all cannula models. By incorporating the time-varying pressure boundary for the tricuspid valve, we can examine variations in the blood flow during the cardiac cycle that are often overlooked in computational studies [41, 42]. Open-tip cannulas generate a high-velocity jet directed toward the cavoatrial junction and inferior vena cava, with only a small fraction of flow exiting through the side holes. This high-velocity jet directed toward the inferior vena cava increases the likelihood that oxygenated blood will be immediately withdrawn by the drainage cannula. In contrast, closed-tip cannulas produce weaker jets that interact primarily with the superior vena cava wall. The flow is slower and promotes greater flow dispersion in the right atrium, thus reducing momentum toward the drainage cannula. These findings are also reflected in Fig. 3 representing $CaO_2$ content in the right atrium. Oxygenated and deoxygenated blood mix predominantly within the atrium for closed-tip cannulas, whereas in open-tip designs, mixing extends into the inferior vena cava. Overall, these results indicate that closed-tip cannulas can reduce recirculation by more effectively directing blood flow. Interestingly, the degree of recirculation depends on the phase of the cardiac cycle, as shown in Fig. 2. Recirculation is minimal during early and late diastole when the flow toward the tricuspid valve is predominant. However, when the valve is closed, the flow toward the drainage cannula increases, thereby increasing recirculation. This suggests that while recirculation could be reduced, it cannot be completely eliminated because of the periodic closure of the tricuspid valve and continuous flow in both cannulas.

One could argue that, despite the improvements in VV-ECMO efficacy, closed-tip cannulas may carry a higher risk of complications, such as thrombosis and hemolysis. In the case of closed-tip cannulas, thrombosis could occur near the cannula cap, where flow oscillations may be triggered. According to our results, closed-tip designs exhibited higher cannula wall-associated thrombotic metrics than the standard open-tip cannula, as shown in Fig. 5. An optimal design appears to require a balance between the tip-to-hole distance (excessive distance promotes blood swirling at low flows) and hole spacing (closely spaced holes create turbulence through interference), since differences in thrombosis-related metrics were observed among closed-tip cannula designs and ECMO flows. Nevertheless, thrombosis-related metrics were consistently lower at the cannula wall than within the surrounding computational domain, an aspect that should be considered when interpreting the findings. In the computational domain, none of the designs showed a pronounced increase compared to the standard cannula, although slight increases were observed at the highest ECMO flow, likely due to reduced inflow blood velocity into the right atrium caused by closed-tip cannula designs, as shown in Fig. 2. Interestingly, in most designs and ECMO flows, elevated thrombotic-related metrics were observed in the right atrial appendage (RAA), as shown in Fig. 6. While VV-ECMO patients are known to be at risk of venous thrombosis, particularly within the circuit and oxygenator [12], the RAA has not previously been identified as a potential site of thrombus formation and it may warrant further clinical investigation. Hemolysis occurs in regions of high shear stress and depends on both stress magnitude and exposure time. In closed-tip cannulas, areas at risk of hemolysis are most likely near the cannula tip, where high-velocity flow impinges on the cap and locally elevates shear stress. As shown in Fig. 5, no significant differences were observed for closed-tip cannulas compared to the standard cannula, indicating that these localised effects do not substantially alter the overall hemolysis-related metrics, either at the cannula wall or within the computational domain.

As mentioned, no universally accepted CFD-derived thresholds for thrombosis and hemolysis exist, and thus the clinical relevance of the observed results should be further validated in experimental and in vivo studies before definitive conclusions can be drawn. Nevertheless, among all tested designs, the 3Rx4H-PerfCap configuration appears to be the most promising candidate for further investigation. It improves VV-ECMO performance by reducing recirculation and enhancing oxygen delivery while also mitigating the thrombosis-related metrics associated with closed-tip geometries, without adversely affecting hemolysis-related metrics.

Clinically, fully closed-tip cannulas can be more challenging to deploy, as the absence of a distal opening complicates guidewire insertion and makes advancement or repositioning more difficult compared to open-tip designs. A most simple approach would be to keep the cannula in a standard open configuration during insertion and advance it over the guidewire. Once correctly positioned, a sealing mechanism at the cannula tip could then be activated. This would also allow standard echocardiographic methods to be used to confirm proper cannula positioning [9]. In the case of the 3Rx4H-PerfCap cannula, which showed the most favourable overall performance, side holes near the distal cap may accommodate an insertion device and help mitigate challenges associated with the sealing mechanism.

While this study provides interesting insights, several limitations should be mentioned. All simulations were conducted using a single patient model, using only the femoro-jugular cannulation strategy and fixed cannula positions, which may restrict the generalizability of the findings. In addition, the conditions investigated represent only a subset of those seen in clinical practice. A more systematic evaluation of physiological parameters, such as cardiac output [17] and heart rate [16], would enable a more comprehensive assessment of the impact of different cannula designs on VV-ECMO performance. Furthermore, vein compliance was not incorporated, and blood was assumed to behave as a Newtonian fluid, both simplifications of the complex hemodynamic properties typically considered in computational studies [17, 18, 42]. Our modelling pipeline focuses just on the right atrium and venous system, excluding the oxygenator and the full VV-ECMO circuit. As a result, while oxygen transport is approximated using an advection–diffusion approach, carbon dioxide clearance and complete gas exchange processes are not explicitly represented, limiting the physiological completeness of the simulation. Moreover, the assessment of thrombosis and hemolysis was based on computational parameters derived from simulations, which may not fully capture all in vivo factors influencing clot formation and blood damage. In addition, the lack of standardised evaluation methods makes cross-study comparisons challenging and requires careful interpretation of the results. Despite all simplifications, this study demonstrates that patient-specific simulations can provide valuable insights into blood flow dynamics, allowing systematic evaluation and optimization of existing and novel cannula designs. Although some of the proposed cannula designs might require alternative insertion strategies and more thorough evaluation, and might even prove unsuitable for clinical use, this approach offers a cost-effective and ethically responsible platform to investigate VV-ECMO performance, reduce reliance on animal experiments, and guide future clinical studies.

## Conclusion

In this study, we used computational modeling to investigate how different return cannula designs affect VV-ECMO performance. We compared open-tip cannulas currently in clinical use with conceptual closed-tip designs across three different ECMO flow rates. Our findings suggest that while cannula design refinements may offer limited benefits when VV-ECMO contributes only modestly to gas exchange, they become increasingly valuable when patients depend primarily on VV-ECMO due to severely compromised native lung function. In particular, closed-tip cannulas can reduce recirculation and improve oxygen delivery, especially at higher ECMO flow rates while maintaining comparable CFD-derived metrics related to thrombogenic potential and hemolysis.

## Supplementary Information

Supplementary material 1.

Supplementary material 2.

Supplementary material 3.

Supplementary material 4.

Supplementary material 5.

Supplementary material 6.

Supplementary file 7.

## Data Availability

The dataset supporting the conclusions of this article, as well as the codes developed and used for the analyses, are available from the corresponding author upon request.

## References

[1] Abrams D, Bacchetta M, Brodie D (2014) Recirculation in venovenous extracorporeal membrane oxygenation. ASAIO J 61(2):115–121. 10.1097/mat.0000000000000179 10.1097/MAT.0000000000000179 25423117

[2] Nunes LB, Mendes PV, Hirota AS, Barbosa EV, Maciel AT, Schettino GPP, Costa ELV, Azevedo LCP, Park M (2014) Severe hypoxemia during veno-venous extracorporeal membrane oxygenation: exploring the limits of extracorporeal respiratory support. Clinics 69(3):173–178. 10.6061/clinics/2014(03)05 24626942 10.6061/clinics/2014(03)05 PMC3935134

[3] Xie A, Yan TD, Forrest P (2016) Recirculation in venovenous extracorporeal membrane oxygenation. J Crit Care 36:107–110. 10.1016/j.jcrc.2016.05.027 27546757 10.1016/j.jcrc.2016.05.027

[4] MacLaren G (2022) Extracorporeal Life Support, The ELSO Red Book, 6th edn.

[5] Palmér O, Palmér K, Hultman J, Broman M (2016) Cannula design and recirculation during venovenous extracorporeal membrane oxygenation. ASAIO J 62(6):737–742. 10.1097/mat.0000000000000440 27660904 10.1097/MAT.0000000000000440 PMC5098462

[6] Gehron J, Bandorski D, Mayer K, Böning A (2023) The impact of recirculation on extracorporeal gas exchange and patient oxygenation during veno-venous extracorporeal membrane oxygenation-results of an observational clinical trial. J Clin Med 12(2):416. 10.3390/jcm12020416 36675344 10.3390/jcm12020416 PMC9866780

[7] Pooth J, Förster JK, Benk C, Diel P, Brixius SJ, Maier S, Supady A, Wengenmayer T, Staudacher DL, Haimerl G, Czerny M, Benk J (2025) Impact of cannulation strategy and extracorporeal blood flow on recirculation during Veno-Venous extracorporeal membrane oxygenation. Artif Organs 49(6):1012–1020. 10.1111/aor.14961 39868656 10.1111/aor.14961 PMC12120813

[8] Hart JP, Davies MG (2024) Vascular complications in extracorporeal membrane oxygenation–a narrative review. J Clin Med 13(17):5170. 10.3390/jcm13175170 39274383 10.3390/jcm13175170 PMC11396245

[9] Hussey PT, Von Mering G, Nanda NC, Ahmed MI, Addis DR (2022) Echocardiography for extracorporeal membrane oxygenation. Echocardiography 39(2):339–370. 10.1111/echo.15266 34997645 10.1111/echo.15266 PMC9195253

[10] Tanaka D, Pitcher HT, Cavarocchi N, Hirose H (2015) Migrated Avalon Veno-Venous Extracorporeal membrane oxygenation cannula: How to adjust without interruption of flow. J Card Surg 30(11):865–868. 10.1111/jocs.12629 26358888 10.1111/jocs.12629

[11] Bukova M, Schumacher T, Mantl M, Funken D, Hoeffler K, Koeditz H, Kaussen T, Tiedge S, Optenhoefel J, Boehne M (2025) Factors influencing recirculation in Veno-Venous extracorporeal membrane oxygenation: insights from a controlled bench study. ASAIO J 72(1):56–64. 10.1097/mat.0000000000002465 40386968 10.1097/MAT.0000000000002465

[12] Nunez JI, Gosling AF, O’Gara B, Kennedy KF, Rycus P, Abrams D, Brodie D, Shaefi S, Garan AR, Grandin EW (2021) Bleeding and thrombotic events in adults supported with venovenous extracorporeal membrane oxygenation: an elso registry analysis. Intensive Care Med 48(2):213–224. 10.1007/s00134-021-06593-x 34921625 10.1007/s00134-021-06593-x PMC9178906

[13] Appelt H, Philipp A, Mueller T, Foltan M, Lubnow M, Lunz D, Zeman F, Lehle K (2020) Factors associated with hemolysis during extracorporeal membrane oxygenation (ecmo)–comparison of va- versus vv ecmo. PLoS ONE 15(1):0227793. 10.1371/journal.pone.0227793 10.1371/journal.pone.0227793 PMC6984694 31986168

[14] Edinger F, Zajonz T, Mayer N, Schmidt G, Schneck E, Sander M, Koch C (2024) A novel model of venovenous extracorporeal membrane oxygenation in rats with femoral cannulation and insights into hemodynamic changes. Biomedicines 12(8):1819. 10.3390/biomedicines12081819 39200283 10.3390/biomedicines12081819 PMC11351971

[15] Leoni M, Szasz J, Meier J, Gerardo-Giorda L (2022) Blood flow but not cannula positioning influences the efficacy of Veno-Venous ECMO therapy. Sci Rep. 10.1038/s41598-022-23159-z 36470881 10.1038/s41598-022-23159-z PMC9722702

[16] Conrad SA, Wang D (2020) Evaluation of recirculation during venovenous extracorporeal membrane oxygenation using computational fluid dynamics incorporating Fluid-Structure interaction. ASAIO J 67(8):943–953. 10.1097/mat.0000000000001314 10.1097/MAT.0000000000001314 PMC8318564 33315664

[17] Parker LP, Marcial AS, Brismar TB, Broman LM, Wittberg LP (2024) In silico parametric analysis of femoro-jugular venovenous ECMO and return cannula dynamics. Med Eng Phys 125:104126. 10.1016/j.medengphy.2024.104126 38508803 10.1016/j.medengphy.2024.104126

[18] Xi Y, Li Y, Wang H, Wang X, Li J, Ji B, Chen Z (2025) The impact of venovenous extracorporeal membrane oxygenation cannulation configuration on hemodynamic characteristics and risks of hemolysis. Ann Biomed Eng. 10.1007/s10439-025-03862-4 41076494 10.1007/s10439-025-03862-4

[19] Parker LP, Fiusco F, Rorro F, Marcial AS, Brismar TB, Broman LM, Wittberg LP (2024) Venovenous extracorporeal membrane oxygenation drainage cannula performance: From generalized to patient-averaged vessel model. Phys Fluids. 10.1063/5.0212546

[20] Azimi M, Liao S, Vatani A, Burrell A, Gregory SD (2022) Improved flow dynamics of extracorporeal membrane oxygenation via design modification of Dual-Lumen cannulas. ASAIO J 68(11):1358–1366. 10.1097/mat.0000000000001669 35184087 10.1097/MAT.0000000000001669

[21] Wong ZY, Azimi M, Khamooshi M, Wickramarachchi A, Burrell A, Gregory SD (2024) The impact of small movements with dual lumen cannulae during venovenous extracorporeal membrane oxygenation: a computational fluid dynamics analysis. Comput Methods Programs Biomed 250:108186. 10.1016/j.cmpb.2024.108186 38692252 10.1016/j.cmpb.2024.108186

[22] Xi Y, Li Y, Wang H, Wang X, Feng W, Chen Z (2025) Effect of structural changes in extracorporeal membrane oxygenation return cannulas on hemodynamic performance and blood damage associated with cannulation. Ann Biomed Eng. 10.1007/s10439-025-03720-3 40167865 10.1007/s10439-025-03720-3

[23] Ecls ELSO-EA. Registry Dashboard | ECMO | Extracorporeal Membrane Oxygenation. https://elso.org/registry/elsoliveregistrydashboard.aspx

[24] Khalili E, Daversin-Catty C, Olivares AL, Mill J, Camara O, Valen-Sendstad K (2024) On the importance of fundamental computational fluid dynamics toward a robust and reliable model of left atrial flows. Int J Numer Methods Biomed Eng 40(4):3804. 10.1002/cnm.3804 10.1002/cnm.3804 38286150

[25] Dirkes N, Key F, Behr M (2024) Eulerian formulation of the tensor-based morphology equations for strain-based blood damage modeling. Comput Methods Appl Mech Eng 426:116979. 10.1016/j.cma.2024.116979

[26] Fedorov A, Beichel R, Kalpathy-Cramer J, Finet J, Fillion-Robin J-C, Pujol S, Bauer C, Jennings D, Fennessy FM, Sonka M et al (2012) 3d slicer as an image computing platform for the quantitative imaging network. Magn Reson Imaging 30(9):1323–1341 22770690 10.1016/j.mri.2012.05.001 PMC3466397

[27] Tonna JE, Abrams D, Brodie D, Greenwood JC, Mateo-Sidron JAR, Usman A, Fan E (2021) Management of adult patients supported with venovenous extracorporeal membrane oxygenation (VV ECMO): Guideline from the Extracorporeal Life Support Organization (ELSO). ASAIO J 67(6):601–610. 10.1097/mat.0000000000001432 33965970 10.1097/MAT.0000000000001432 PMC8315725

[28] Salome Consortium: SALOME Platform. https://www.salome-platform.org/. Accessed: July 2025 (2025)

[29] Pooboni SK, Gulla KM (2020) Vascular access in ECMO. Indian J Thorac Cardiovasc Surg 37(S2):221–231. 10.1007/s12055-020-00999-w 33967445 10.1007/s12055-020-00999-w PMC8062664

[30] Getinge Group: HLS Cannulae. (2023). Getinge Group. Accessed: 2025-07-14. https://www.getinge.com/int/products/hls-cannulae/

[31] Medtronic: Bio-Medicus NextGen cannulae for cardiac surgery. https://www.medtronic.com/me-en/healthcare-professionals/products/cardiovascular/cannulae/bio-medicus-nextgen.html

[32] IT’IS Foundation: Viscosity – IT’IS Database for Thermal and Electromagnetic Parameters of Biological Tissues (2024). https://itis.swiss/virtual-population/tissue-properties/database/viscosity/

[33] Yartsev A. The cardiac cycle. https://derangedphysiology.com/main/cicm-primary-exam/cardiovascular-system/Chapter-003/cardiac-cycle

[34] Loosen G, Conrad AM, Hagman M, Essert N, Thiel M, Luecke T, Krebs J (2021) Transpulmonary thermodilution in patients treated with veno-venous extracorporeal membrane oxygenation. Ann Intensive Care. 10.1186/s13613-021-00890-w 34213674 10.1186/s13613-021-00890-w PMC8249841

[35] Baratta I, Dean J, Dokken JS, Habera M, Hale JS, Richardson C, Rognes ME, Scroggs MW, Sime N, Wells GN (2023) Supplementary Material. DOLFINx: The next generation FEniCS problem solving environment. 10.5281/zenodo.10026723

[36] Johann Radon Institute for Computational and Applied Mathematics (RICAM): Radon1 High-Performance Computing Cluster. https://www.oeaw.ac.at/ricam/hpc

[37] Doyle D (1989) Arterial saturation from PO2 (SAT), in Computer Programs in Clinical and Laboratory Medicine, pp 45–47. 10.1007/978-1-4612-3576-7_8

[38] Giersiepen M, Wurzinger LJ, Opitz R, Reul H (1990) Estimation of shear stress-related blood damage in heart valve prostheses - in vitro comparison of 25 aortic valves. Int J Artif Organs 13(5):300–306. 10.1177/039139889001300507 2365485

[39] Bludszuweit C (1995) Model for a general mechanical blood damage prediction. Artif Organs 19(7):583–589. 10.1111/j.1525-1594.1995.tb02385.x 8572956 10.1111/j.1525-1594.1995.tb02385.x

[40] Schmidt M, Tachon G, Devilliers C, Muller G, Hekimian G, Bréchot N, Merceron S, Luyt CE, Trouillet J-L, Chastre J, Leprince P, Combes A (2013) Blood oxygenation and decarboxylation determinants during venovenous ECMO for respiratory failure in adults. Intensive Care Med 39(5):838–846. 10.1007/s00134-012-2785-8 23291732 10.1007/s00134-012-2785-8

[41] Parker LP, Marcial AS, Brismar TB, Broman LM, Wittberg LP (2023) Hemodynamic and recirculation performance of dual lumen cannulas for venovenous extracorporeal membrane oxygenation. Sci Rep 13(1):7472. 10.1038/s41598-023-34655-1 37156961 10.1038/s41598-023-34655-1 PMC10167322

[42] Hörwing H, Parker L, Svensson-Marcial A, Brismar TB, Broman LM, Wittberg LP (2025) Hemodynamics in femoro-femoral venovenous extracorporeal membrane oxygenation using large eddy simulations. Sci Rep 15(1):35229. 10.1038/s41598-025-22403-6 41068411 10.1038/s41598-025-22403-6 PMC12511417

