# Supplementary material for: Digital performance testing reveals how redesigned return cannulas can enhance VV-ECMO performance
Source: Intensive Care Med Exp. 2026 Jun 15;14:75. doi: 10.1186/s40635-026-00927-z (PMC13269575; doi:10.1186/s40635-026-00927-z)
Supplement: Supplementary file 2 — Supplementary material 2. [file 40635_2026_927_MOESM7_ESM.pdf]

# Supplementary Material: Digital Performance Testing Reveals How Redesigned Return Cannulas Can Enhance VV-ECMO Performance

Beata Ondrusova[1], Argyrios Petras[2], Johannes Szasz[3], Jens Meier[3], Luca Gerardo-Giorda[1,2]*

[1]Institute for Mathematical Methods in Medicine and Data Based Modeling, Johannes Kepler University, Linz, Austria

[2]Johann Radon Institute for Computational and Applied Mathematics (RICAM), Linz, Austria

[3]Kepler University Klinikum, Linz, Austria

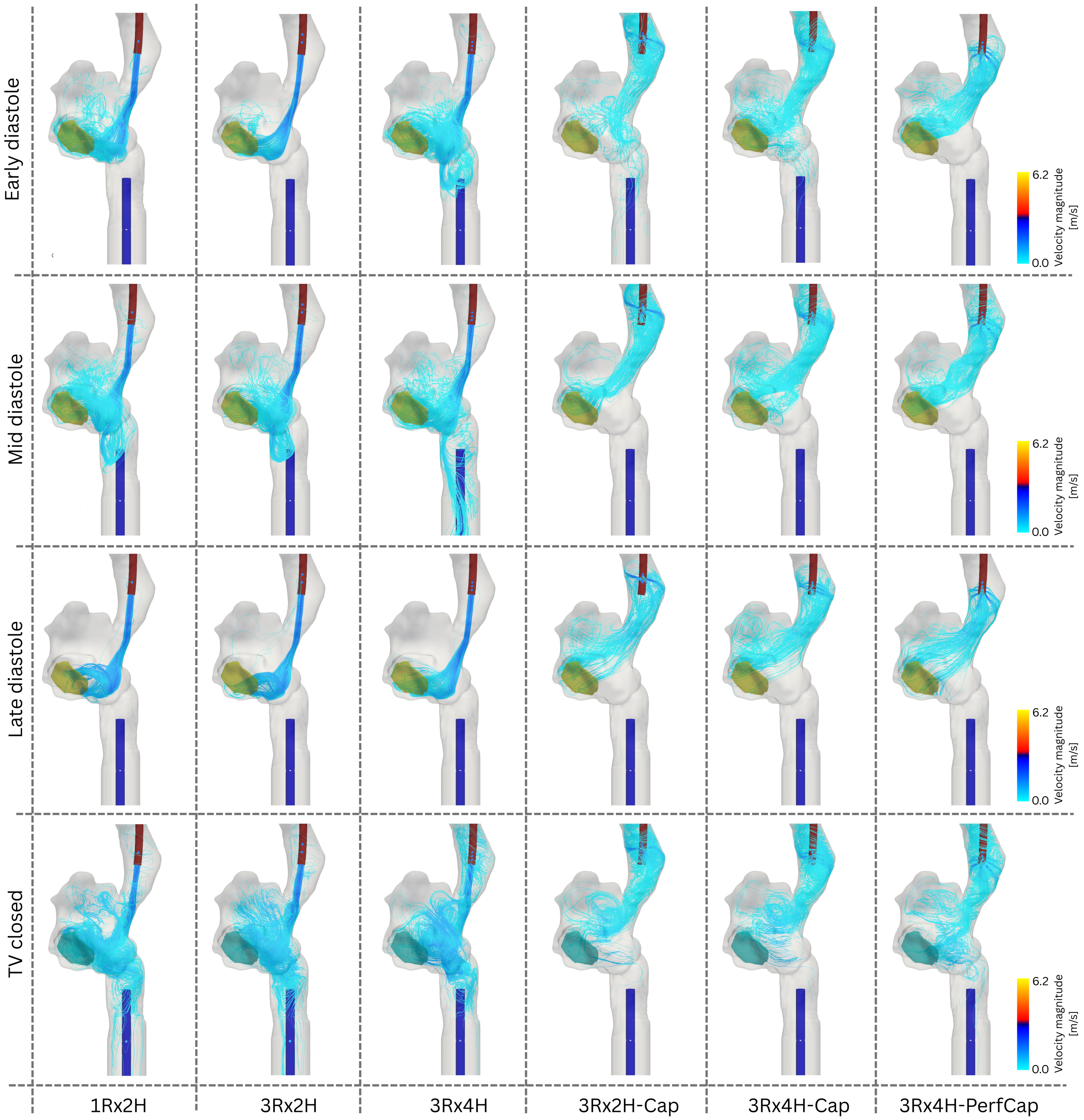


Figure 1: Blood flow from return cannula during different phases of the ventricular cycle (rows) for all cannula designs (columns). The colour of the TV indicates the cardiac cycle phase: yellow when open and blue when closed. The results are depicted for the ECMO flow of 2L/min.

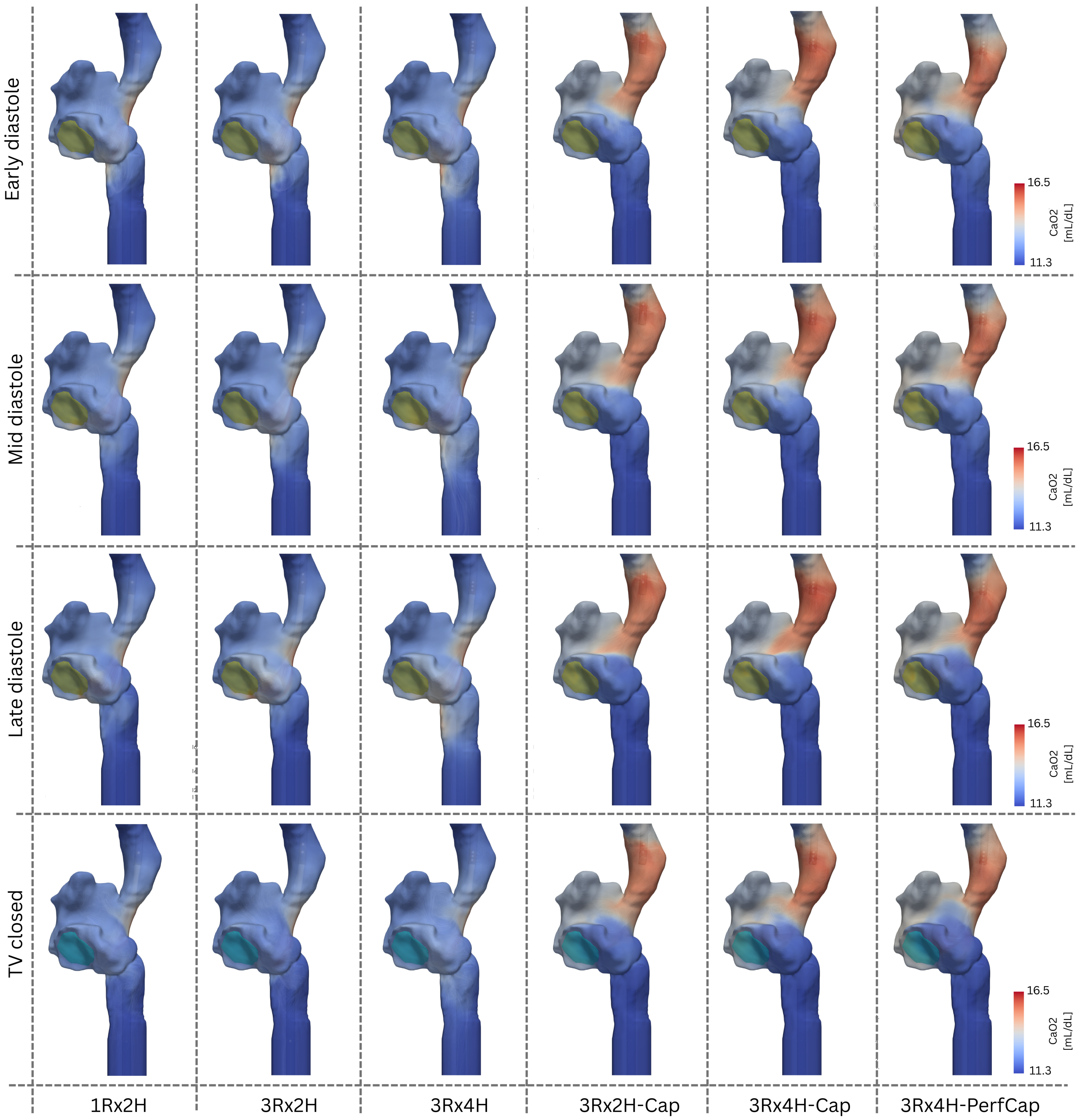


Figure 2: Oxygen content ($CaO_2$) in right atrium during different phases of the ventricular cycle (rows) for all cannula designs (columns). The colour of the tricuspid valve indicates the cardiac cycle phase: yellow when open and blue when closed. The results are depicted for the ECMO flow of 2L/min.

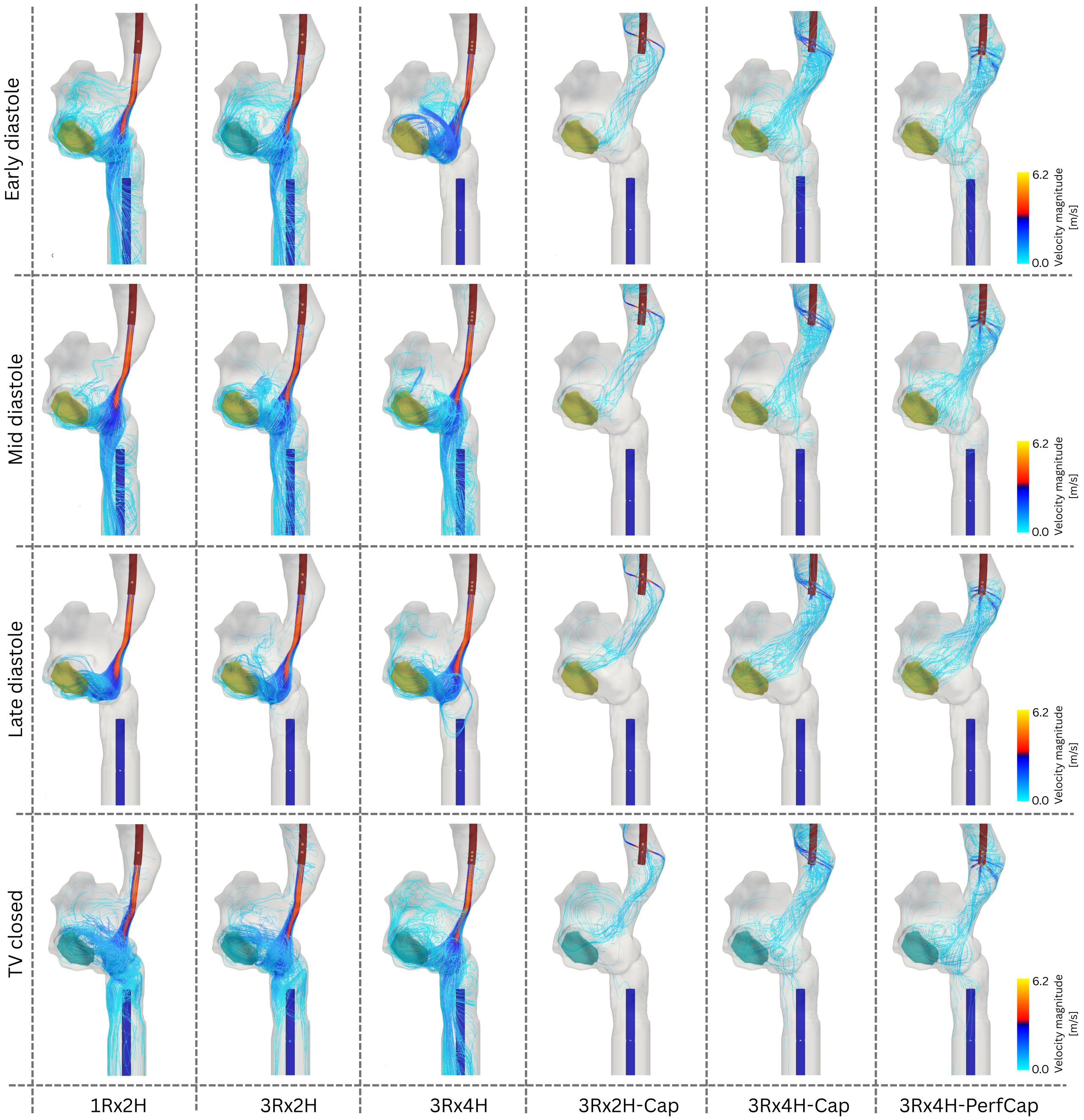


Figure 3: Blood flow from return cannula during different phases of the ventricular cycle (rows) for all cannula designs (columns). The colour of the tricuspid valve indicates the cardiac cycle phase: yellow when open and blue when closed. The results are depicted for the ECMO flow of 6L/min.

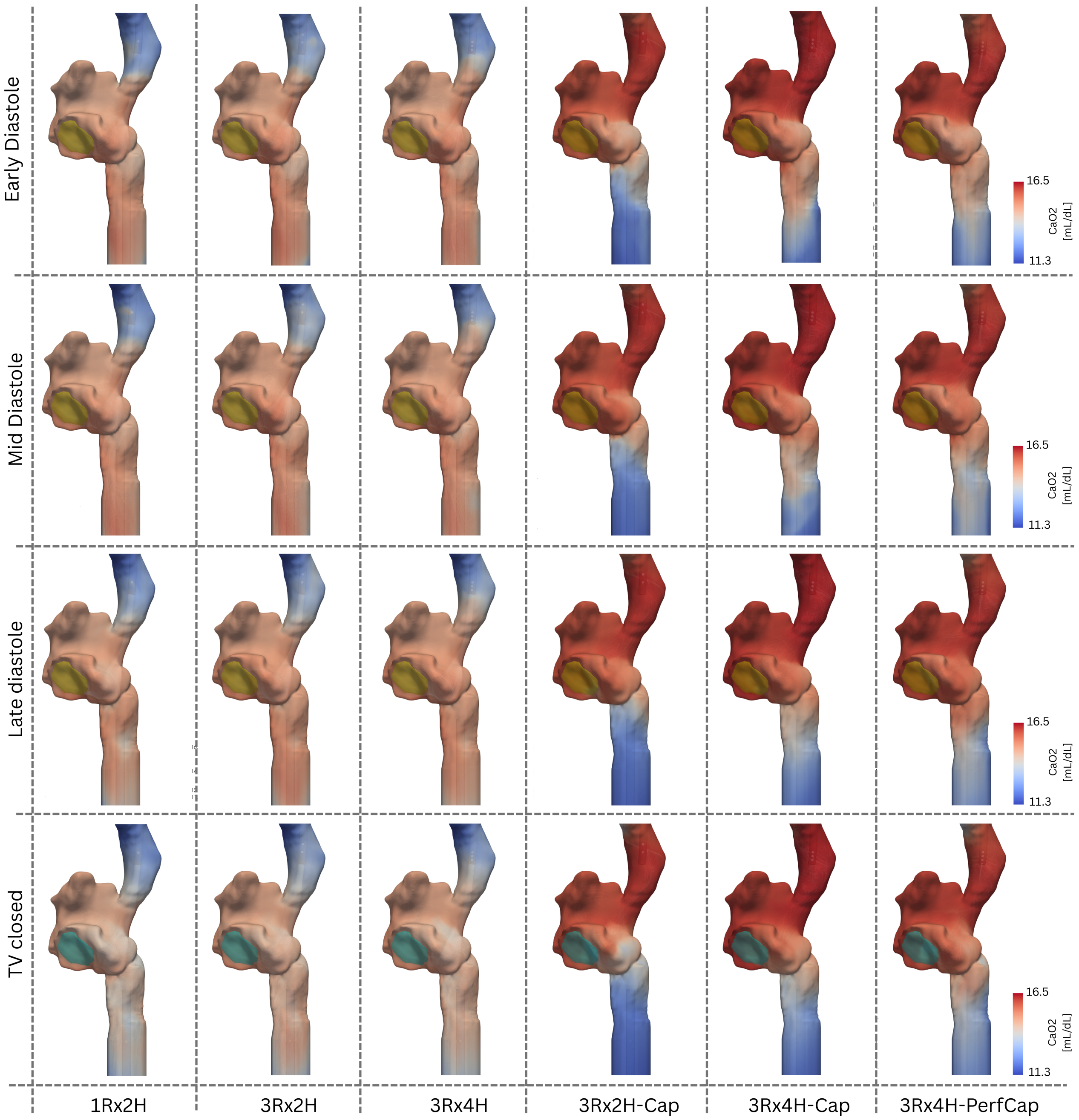


Figure 4: Oxygen content ($CaO_2$) in right atrium during different phases of the ventricular cycle (rows) for all cannula designs (columns). The colour of the tricuspid valve indicates the cardiac cycle phase: yellow when open and blue when closed. The results are depicted for the ECMO flow of 6L/min.

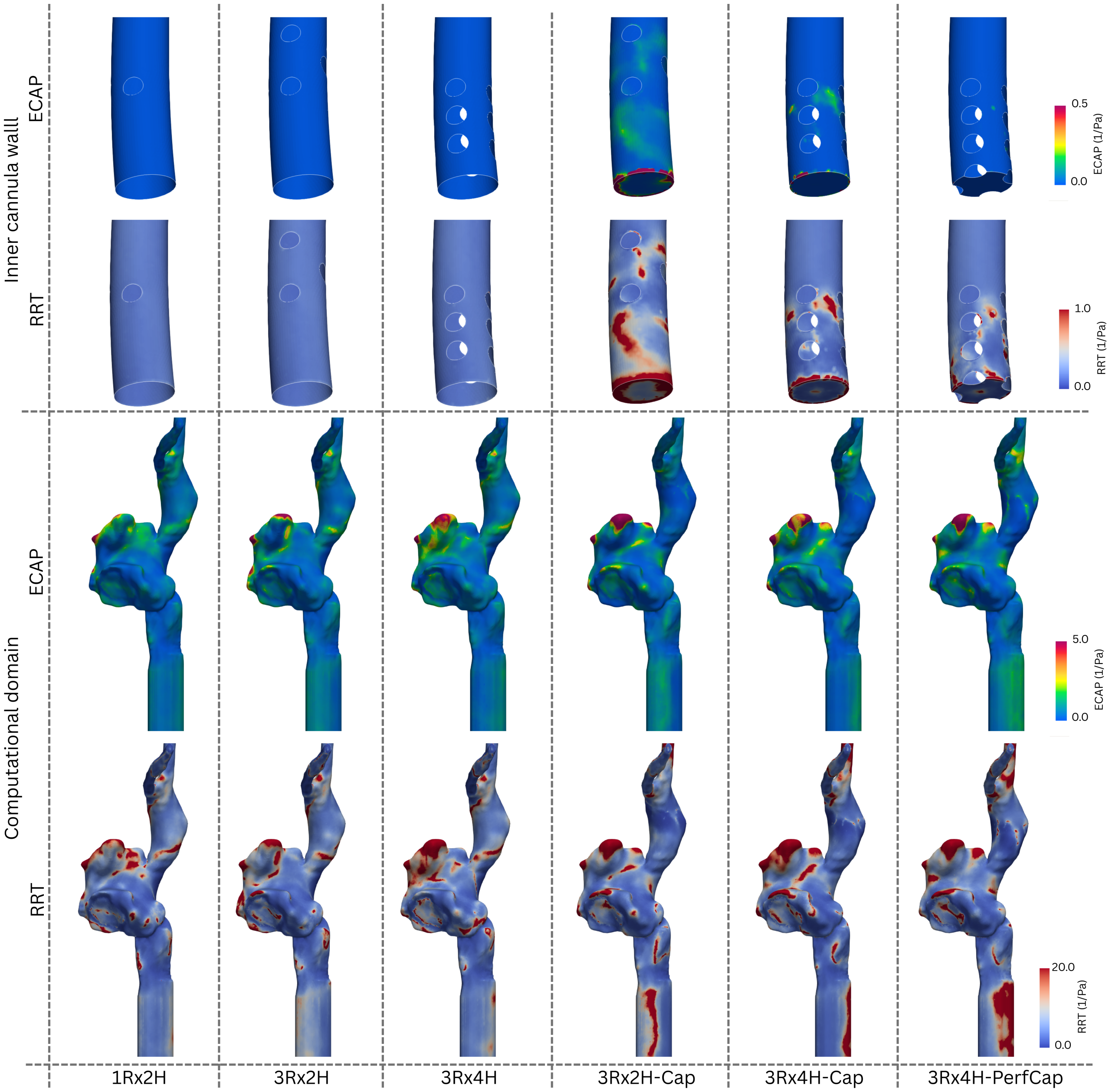


Figure 5: Endothelial cell activation potential (ECAP) and relative residence time (RRT) depicted for the inner wall of the returning cannula and the computational domain consisting of walls of the veins and right atrium. Colorbar limits are set separately for the cannula and the computational domain because of differences in their respective values. The results are depicted for the ECMO flow of 2L/min.

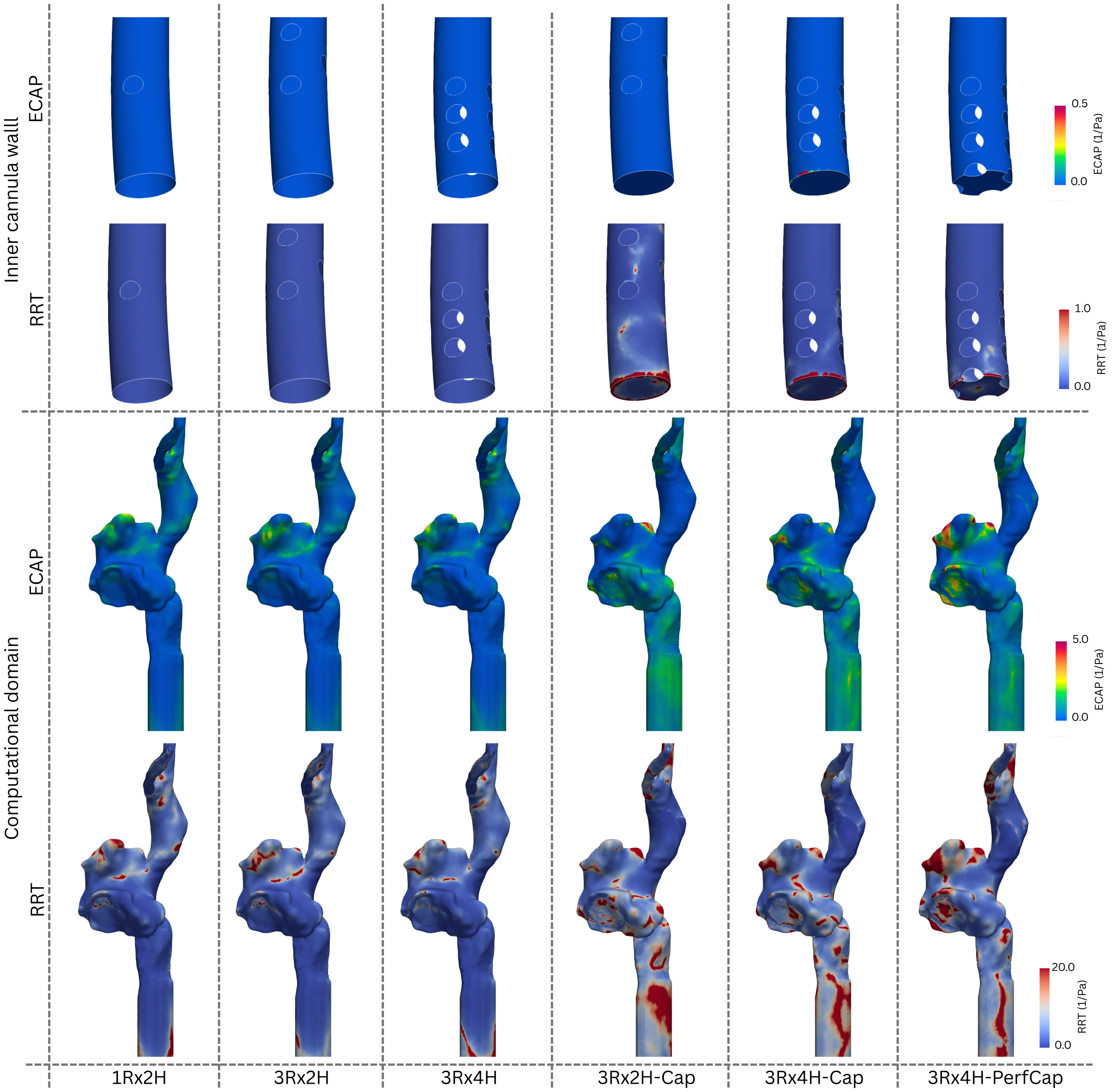


Figure 6: Endothelial cell activation potential (ECAP) and relative residence time (RRT) depicted for the inner wall of the returning cannula and the computational domain consisting of walls of the veins and right atrium. Colorbar limits are set separately for the cannula and the computational domain because of differences in their respective values. The results are depicted for the ECMO flow of 6L/min.

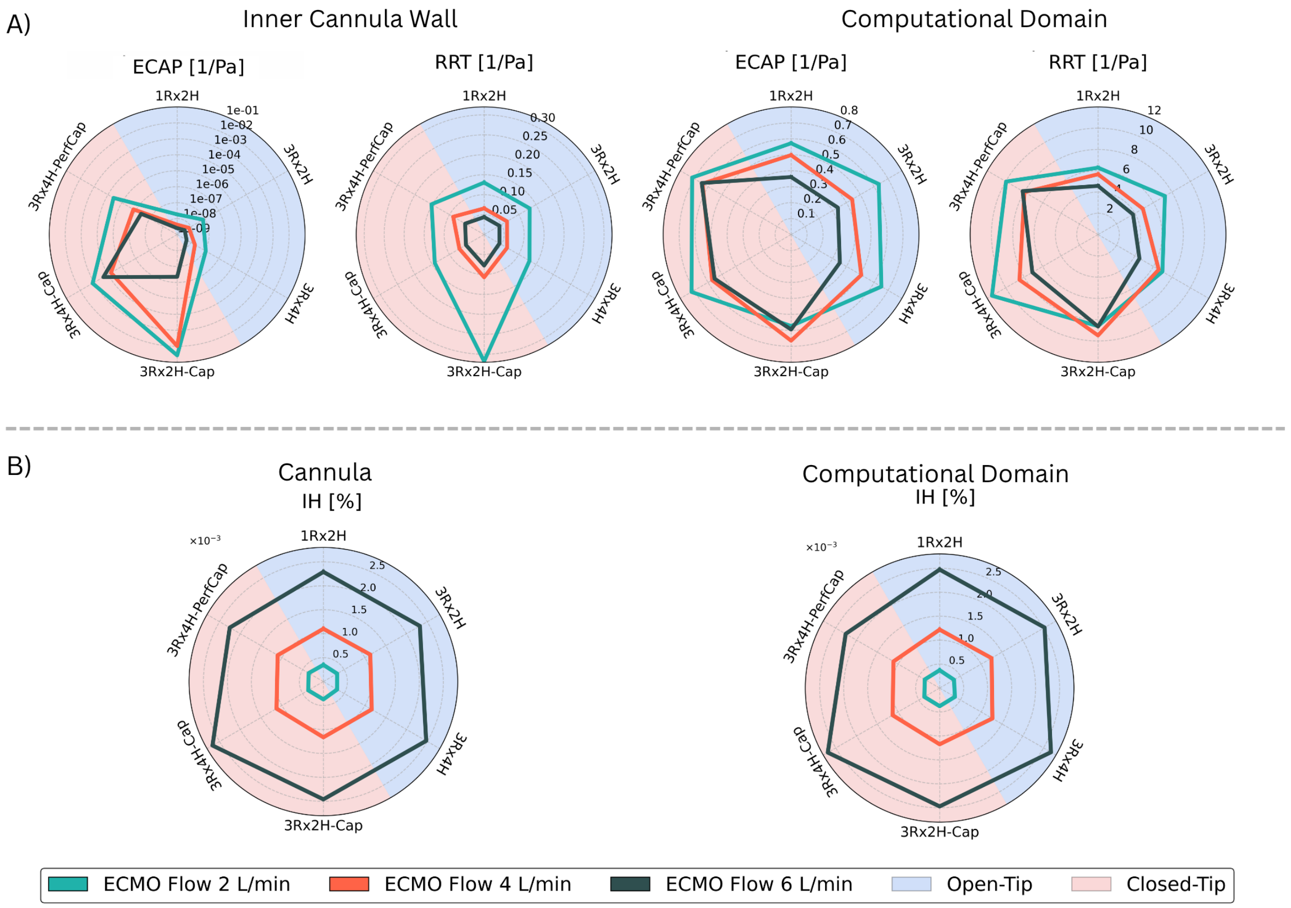


Figure 7: A) Radar plots depicting mean values of endothelial cell activation potential ($ECAP$) and relative residence time ($RRT$) computed for the inner wall of the returning cannula and the computational domain consisting of walls of the veins and right atrium. B) Radar plots depicting the mean index of hemolysis (IH) for pathlines from the returning cannula and for pathlines in the entire computational domain, including the walls of the veins and right atrium. For all radar plots, cannula designs are represented on separate axes radiating from a central point, with different ECMO flow rates indicated by distinct colors.
